# Standardization of Antigen-Emulsion Preparations for the Induction of Autoimmune Disease Models

**DOI:** 10.3389/fimmu.2022.892251

**Published:** 2022-06-13

**Authors:** Louise M. Topping, Laura Romero-Castillo, Vilma Urbonaviciute, Hans Bolinsson, Felix I. Clanchy, Rikard Holmdahl, B. Thomas Bäckström, Richard O. Williams

**Affiliations:** ^1^ Kennedy Institute of Rheumatology, Nuffield Department of Orthopaedics, Rheumatology and Musculoskeletal Sciences, University of Oxford, Oxford, United Kingdom; ^2^ Medical Inflammation Research, Department of Medical Biochemistry and Biophysics, Karolinska Institute, Stockholm, Sweden; ^3^ Department of Food Technology, Engineering and Nutrition, Lund University, Lund, Sweden; ^4^ The Rausing Laboratory, Division of Neurosurgery, Department of Clinical Sciences, Lund University, Lund, Sweden; ^5^ Department of Autoimmunity, BTB Emulsions AB, Malmo, Sweden; ^6^ Botnar Research Centre, Nuffield Department of Orthopaedics, Rheumatology and Musculoskeletal Sciences, University of Oxford, Oxford, United Kingdom

**Keywords:** autoimmunity, animal disease models, EAE, CIA, antigen emulsions, Complete Freund’s Adjuvant, emulsification

## Abstract

Autoimmune murine disease models are vital tools for identifying novel targets and finding better treatments for human diseases. Complete Freund’s adjuvant is commonly used to induce disease in autoimmune models, and the quality of the adjuvant/autoantigen emulsion is of critical importance in determining reproducibility. We have established an emulsification method using a standard homogenizer and specially designed receptacle. Emulsions are easy to prepare, form stable and uniform water-in-oil particles, are faster to make than the traditional syringe method, use less material and are designed to fill syringes with ease. In the present study, we have validated the emulsions for induction of experimental autoimmune encephalitis, collagen II induced arthritis, antigen induced arthritis, and delayed type hypersensitivity models. These models were induced consistently and reproducibly and, in some cases, the new method outperformed the traditional method. The method described herein is simple, cost-effective and will reduce variability, thereby requiring fewer animals for *in vivo* research involving animal models of autoimmune disease and in vaccine development.

## Introduction

Mouse models of autoimmune disease remain vital tools for researchers to investigate pathogenesis as well as develop novel therapies. As many autoimmune diseases, including rheumatoid arthritis (RA) and multiple sclerosis (MS), continue to have no cure and complex etiology, animal models of disease are used in preclinical research.

For a number of autoimmune diseases, no spontaneous animal models of disease exist, and disease is induced by immunizing animals with autoantigens emulsified in adjuvants to break tolerance (1). For the study of MS, experimental autoimmune encephalitis (EAE) is a widely used inducible model, causing MS-like disease in animals immunized with spinal cord homogenate or myelin-derived proteins or peptides, mixed with immunostimulants (2–4). Similarly, a commonly used animal model for RA is collagen-induced arthritis (CIA), in which mice are immunized with collagen II (Col2) proteins, derived from different animal species, alongside adjuvants to induce a joint specific pathology (5). Pristane-induced arthritis (PIA) is a rat model for RA that can be induced in susceptible mouse strains by two I.P. injections of Pristane (2,6,10,14 tetramethylpentadecane) (6, 7). Inducible models continue to be favored by researchers due to the shared characteristics with human disease, including the loss of tolerance, induction of autoreactive T cells and the generation of autoantibodies.

The most commonly used adjuvant to induce autoimmune diseases like EAE and CIA is Complete Freund’s Adjuvant (CFA), a mineral oil containing dried *Mycobacterium tuberculosis* bacterial fragments. Animals will not develop disease unless a homogeneous water-in-oil emulsion is prepared by mixing auto-antigens dissolved in aqueous buffer with CFA. This adjuvant was first was first reported in 1942 (8, 9) and has been used in animals for decades for the induction of autoimmune models, delayed-type hypersensitivity models as well as the generation of monoclonal and polyclonal antibodies (10), vaccine adjuvant for HIV seropositive individuals (11) and cancer vaccines (12). Although it has been used for decades to induce disease, the exact mode of action of CFA isn’t completely understood. However, early studies suggested that CFA is able to prolong the local half-life of antigens at the site of injection (13), as well as enhance phagocytosis (14) and causes activation of the innate immune system, which in turn directs and orchestrates the development of antigen specific T and B cells (10).

Although CFA induces a strong immune response even in the absence of intentionally added autoantigens such as myelin or cartilage, several autoimmune models of disease are difficult to induce consistently. Marked differences between mouse strains result in differences in responses to autoimmune inductions, reflecting the genetic variation associated with autoimmune susceptibility (15, 16). Although the optimal inbred mouse strain for each disease model has been identified, models such as CIA and EAE often still do not reach 100% disease penetrance with a high degree of variability in severity (17, 18). Therefore, consistency in the preparation of antigen emulsions and induction of disease are of utmost importance to ensure maximum disease penetrance and reproducibility. Nonetheless, there are currently no standardized protocols for the preparation of antigen-emulsions for the induction of autoimmune disease, with laboratories often using a manual syringe-based method to produce their emulsions.

Another field where water-in-oil emulsions has become been increasingly potential is within human therapeutic cancer vaccines. Vaccine adjuvants, such as Montanide™ ISA 51, has shown to be a promising adjuvants for cancer vaccines (19). A standardizes protocol to prepare water-in oil emulsions in the clinic would have a huge impact, not only in clinical trials but also when treating patients with life threatening diseases such as a number of cancers.

Herein, we present a standardized homogenization method using a homogenizer and specially designed consumables, namely POWER-Kit. POWER-Kit™ based emulsions are easy to prepare, faster to make than the traditional syringe method, use less antigen and CFA compared to the syringe method, and syringes can be loaded with ease and minimal waste. In the present study, we validate the use of POWER-Kit™ emulsions for the first time to demonstrate the consistent and reproducible induction of autoimmune diseases and delayed-type hypersensitivity (DTH).

## Materials and Methods

### POWER Emulsification

#### Equipment and Materials

Minilys Personal Homogenizer from Bertin Instruments (catalogue no P000673-MLYS0-A, Montigny-le-Bretonneux, France), POWER-Kit™ (BTB Emulsions AB, Malmö, Sweden catalogue no: PK-M www.btbemulsions.com) and Injekt^®^-F Solo 2-piece fine dosage syringe 1ml syringes (B Braun, Melsungen, Germany).

The detailed procedure can be found at https://btbemulsions.com/technology. The volumes needed for all the reagents were calculated and 20% was added for each reagent to compensate for dead-space losses in the tube and syringes. The ratio of aqueous solution/adjuvant was always kept 1:1 (v/v). If other syringes than B Braun’s Injekt^®^-F Solo 1ml syringes were used, 30-40% extra emulsion were prepared due to higher losses. All the necessary reagents were placed on ice. The reagents were added to the tube in the following order: 1) PBS without Ca^2+^/Mg^2+^, 2) the antigen and 3) lastly CFA. The lid was then tightened firmly and then the tube was shaken vigorously by hand for 5 seconds. Thereafter, it was place it in the MiniLys Homogenizer, shaken for 60 seconds at the highest speed setting, and then placed and covered by ice for 3 minutes. The procedure was repeated 1-2 times with the same settings. To remove trapped air bubbles and compact the emulsion, the tube was centrifuge for 1 minute at 300*g*. The emulsion was then transferred to B Braun’s Injekt^®^-F Solo syringes. The green plunger from the syringes were removed and saved for later use. The lid on the tube was unscrewed and the plunger from the POWER-Kit™ inserted. It was pushed down so that it just touched the top of the emulsion. The tear off piece at the bottom of the tube was removed and the back end of the B Braun syringe was attached to the tube with a short twist. The POWER-Kit™ plunger was pushed into the tube slowly until the emulsion reached the 0,15ml mark on the syringe. At this stage, the syringe was removed from the tube and a 25G x 16mm (⅝”) hypodermic needle was attached. The saved green plunger from the B Braun syringe was then slowly insert, making sure no air was trapped around the opening. The plunger was further inserted until the emulsion appeared at the end of the needle. This procedure was continued until all syringes were loaded with emulsion.

To compare the POWER-Kit™ method, other emulsions were prepared using either manual mixing, 2-syringe mixing or vortex protocol. For manual mixing, CFA and the aqueous phase (PBS and antigen) were added to a bijous tube and kept on ice. A syringe was used to mix and emulsify by drawing the CFA and aqueous solution into and out of the syringe repeatedly. For the 2-syringe method, CFA and the aqueous phase were loaded into two separate syringes and then connected to a 3 way stop cock (BD Catalogue no 394995). The emulsion was kept cold during the preparation and the syringes were pushed back and forth. For emulsions prepared using a Vortex (Scientific Industries SI™ Vortex-Genie™ 2), CFA, PBS and peptide were added to a 15 ml Falcon tube. The tube was placed upright on the vortex held in place by a piece of silver duct tape. The vortex was turned to maximum setting (3,200 rpm) for 10 minutes. The tube was then put on ice for 3 minutes and then vortexed again for another 10 minutes. A drop-test was performed to confirm that a water-in-oil emulsion had been formed for all methods (data not shown). If the drop test failed, mixing continued and emulsion was retested.

To better assess the quality of produced emulsions, we inspected emulsions using a phase contrast microscope. A tiny drop (10-20μl) of the emulsion was placed on a microscope slide, smeared out with a cover slip and then pushed hard with a circular motion to flatten out the emulsion. The emulsion was then examined under 400x magnification and focused on a field with a monolayer of the emulsion.

### Laser Diffraction Particle Size Analysis

Laser diffraction measures particle size distributions by measuring the angular variation in intensity of light scattered as a laser beam passes through a dispersed particulate sample. Large particles scatter light at small angles and small particles scatter light at larger angles, relative to the direction of the laser beam. The angular scattering intensity data is analyzed to calculate the sizes of the particles responsible for creating the scattering pattern, using e.g. the Mie theory of light scattering. The particle size is reported as a volume equivalent sphere diameter and the particle size distribution (PSD) can be estimated from the primary data.

The particle size measurements of the emulsion, for the storage study, were performed using a Mastersizer 2000 (Malvern Panalytical Ltd, Malvern, UK) equipped with a dispersion unit (Hydro 2000SM). The stirring speed was set to 2000rpm. Diluent granulometrique (Batch L0661, Seppic SA, La Garenne Colombes, France) was used as the dispersant. The sample emulsion was prepared and then stored in a 1ml syringe throughout the course of the study.

Before every measurement, the first few drops from the syringe were discarded. The emulsion was introduced in the dispersion unit directly from the syringe in a single drop, to the lowest achievable volume, and to an obscuration level of 5-8%. Data acquisition was started instantly after dispersion of the emulsion. A general-purpose model was applied, and spherical particles were assumed, for the estimation of particle size distributions. The refractive indices set, for both red and blue lasers, were for the dispersant 1.456 and for the sample 1.33. The absorbance of the refractive index was set to 0.02. Between measurements the emulsion was stored at 4 degrees Celsius. The 25G x 16mm (⅝”) hypodermic needle was exchanged between measurements.

### Mice

For experiments performed at Lund University, all animal procedures were performed according to the practices of the Swedish Board of Animal Research and were approved by the Animal Ethics Committee, Lund-Malmö, Sweden (Permit number: M126-16). Female C57BL/6JRj mice were purchased from Janvier Labs (Le Genest-Saint-Isle, France) and used at 9-11 weeks of age. All mice were maintained at 21°C ± 2°C and 12-hour light/12-hour dark cycle, with food and water available ad libitum.

For experiments performed at the Karolinska Institute, the local ethics committee approved all animal experiments (Stockholms Norra Djurförsöksetiska Nämnd, Stockholm, Sweden). All *in vivo* arthritis experiments were covered by the ethical number N35/16. The C57BL/10.Q (B10.Q) mice has been maintained in the medical inflammation research (MIR) animal house for more than 20 years and has been earlier characterized (20).

For induction of the human Glucose-6-phosphate isomerase (GPI) peptide 325-339-induced arthritis (GPIA), earlier described in (21), C57BL/6J male mice 12-16 week of age were used. For the CIA experiments, male QB mice (B10.Q × BALB/c) F1 were used at 12–16 week of age. BALB/c were purchased from The Jackson Laboratory. For DTH experiments, knock-in HLA-DRB1*0401.hIi.cia9i mice, expressing a functional part of human DRB1*0401 MHC class II molecule as well as humanized invariant chain (hIi) were used (generously provided by Vacara AB, Sweden). In addition, a *Mus musculus* derived chromosomal fragment (*cia9i*), correcting the genome, and which also increases susceptibility to autoimmune disease models was introduced to the strain (22). The strain was purchased from Ozgene. All mice were maintained at 21°C ± 2°C and 12-hour light/12-hour dark cycle, with food and water available ad libitum. The experimental protocol with scoring method has earlier been described in detail (23).

For experiments performed at the Kennedy Institute of Rheumatology, DBA/1 and C57BL/6J were purchased from Envigo (Horst, Netherlands). All experimental procedures were approved by the Ethical Review Process Committee and the UK Home Office, in accordance with the 1986 Animals (Scientific Procedures) Act. For CIA, male DBA/1 mice aged 8-12 weeks were used. For AIA model, male C57BL/6J mice aged 8-10 weeks were used. Mice were housed in ventilated cages, maintained at 21°C ± 2°C and a 12-hour light/12-hour dark cycle, with food and water available ad libitum.

For experiments performed at Redoxis AB, Sweden, all animal procedures were performed according to the practices of the Swedish Board of Animal Research and were approved by the Animal Ethics Committee, Lund, Sweden (Permit number: M79-16). Female C57BL/6JRj and DBA/1 mice were purchased from Janvier Labs (Le Genest-Saint-Isle, France) and used at 9-11 weeks of age. All mice were maintained at 21°C ± 2°C and 12-hour light/12-hour dark cycle, with food and water available ad libitum.

### Experimental Autoimmune Encephalomyelitis

For EAE experiments performed at Lund University, Freund’s adjuvant/antigen emulsions were prepared using POWER-Kit™. Mice were injected subcutaneously at the flank on the back of the mouse with 200µl (100µl on each side) consisting of 50μg mouse MOG_35-55_ peptide (synthesized by Innovagen, Lund, Sweden) emulsified in CFA containing 300μg Mycobacterium tuberculosis H37RA (Difco, Detroit, MI) on day 0. Bordetella pertussis toxin (Sigma-Aldrich, Catalogue no P2980) was injected 80ng/200μl in pertussis toxin buffer (Ca2+/Mg2+ free PBS with the addition of 0,5M NaCl and 0,01% Triton X-100) intraperitoneally (100µl on each side) on day 0 (at least 1,5 hours after immunization) and again after 24 hours. The mice were observed daily for clinical signs and scores were assigned based on the following scale; 0.5 tail not normal, 1 weakness of the tail, 1.5 paralysis in part of the tail, 2 paralysis throughout the tail, 2.5 walking affected, 3 walking affected and weakness in one or two hind legs, 3.5 paralysis in a hind leg, 4 paralysis in a hind leg and partly also in the other hind legs, 4.5 paralysis in both hind legs, 5 paralysis in part of the back part, 5.5 paralysis throughout the back part, 6 paralysis in one of the forelegs. At this stage, the animals were culled according to ethical termination point.

For EAE experiments performed at Redoxis AB, Lund, Sweden (www.redoxis.se), Freund’s adjuvant/antigen emulsions were prepared, either using POWER-Kit™ (see above), or a standard 2-syringe protocol. For the syringe protocol, the oil phase (Freund’s adjuvant) and the water phase (peptide) were loaded into two separate syringes and then connected to a 3 way stop cock (BD Catalogue no 394995). The emulsion was kept cold during the preparation and the phases were pushed through the syringes until a white solid emulsion appeared. A drop-test was performed to confirm that a water-in-oil emulsion had been formed.

Mice (C57BL/6JRj) were injected subcutaneously at the flank on the back of the mouse with 100μl consisting of 200μg mouse MOG_35-55_ peptide (synthesized by Red Glead Discovery, Lund, Sweden) emulsified in CFA containing 300μg Mycobacterium tuberculosis H37RA (Difco, Detroit, MI) on day 0. Bordetella pertussis toxin (Calbiochem, Merck Millipore, catalogue no 516560) was injected (200ng/200μl in PBS) intraperitoneally on day 0 (at least 1,5 hours after immunization) and again after 24 hours. The mice were observed daily for clinical signs and scores were assigned based on the following scale; 1 = tail weakness, 2 = tail paralysis, 3 = tail paralysis and mild waddle, 4 = tail paralysis and severe waddle, 5 = tail paralysis and paralysis of one limb, 6 = tail paralysis and paralysis of a pair of limbs, 7 = tetraparesis or paralysis of three limbs. At this stage, the animal must be culled according to ethical termination points.

### Collagen-Induced Arthritis

For CIA experiments performed at the Kennedy Institute of Rheumatology, immunization was carried out as previously described (24). In brief, bovine type II collagen (bCol2) was purified from articular cartilage and dissolved in 0.1 M acetic acid. bCol2 at 4 mg/ml was emulsified with an equal volume of CFA (BD Biosciences) using manual emulsification method or POWER emulsification method. Male DBA/1 mice aged 8-10 weeks were immunized by subcutaneous injection of 100μl emulsion. After immunization, mice were monitored daily for arthritis. The clinical severity of CIA was scored in each paw as follows: 0=normal, 1=slight swelling and/or erythema, 2=marked swelling, 3=ankylosis. Hindpaw thickness was measured using microcalipers (Kroeplin, Schluchlem, Germany).

For CIA experiments performed at the Karolinska Institute, rat type II collagen (rCol2) was prepared from the Swarm chondrosarcoma, by limited pepsin digestion, and further purified, as previously described (25). The rCol2 was stored at 4°C until used. To induce CIA, DBA/1 mice were injected with 100μg of rCol2 emulsified with an equal volume of CFA (Difco, Detroit, MI, USA) at the base of the tail in a total volume of 100μl. Thirty-five days later, the mice were given a booster injection of 50μg of rCol2 emulsified 1:1 in IFA (Difco, Detroit, MI, USA) in a total volume of 50μl.

For CIA experiments performed at Redoxis AB, Lund, Sweden (www.redoxis.se), DBA/1 mice (10/group) were immunized with 100μg chicken Col2 (Chondrex, Woodinville, WA, USA) emulsified with an equal volume of CFA (Difco, Detroit, MI, USA) using the POWER-Kit™ method. After 21 days, the mice were given a booster injection of 50μg of chicken Col2 emulsified 1:1 in IFA, prepared as above. The clinical severity of CIA was scored in a blinded fashion using a macroscopic scoring system of the four limbs; ranging from 0 to 15 (1 point for each swollen or red toe, 1 point for a swollen or red mid foot digit or knuckle, 5 points for a swollen ankle) resulting in a maximum total score of 60 for each mouse.

To induce GPIA, B10Q mice at the animal facility at the Karolinska Institute were immunized with 10µg of human GPI (hGPI) peptide 325-339 (Biomatik, Wilmington, DE) emulsified with an equal volume of CFA at the base of the tail in a total volume of 100μl (21). Development of clinical arthritis was followed through visual scoring of the animals based on the number of inflamed joints in each paw, starting 2 weeks post-immunization for CIA and day 9-10 post-immunization for GPIA and continuing until the end of the experiment. For both arthritis models, an extended scoring protocol ranging from 1 to 15 for each paw with a maximum score of 60 per mouse was used (23). Each arthritic toe and knuckle were scored as 1, with a possible max score of 60. A score of 5 was given to an arthritic ankle. The mice were examined two to four times per week for maximum 90 days after immunization.

### Antigen Induced Arthritis

AIA was performed at the Kennedy Institute of Rheumatology as previously described (26). Briefly, methylated bovine serum albumin (mBSA) at 2mg/ml was emulsified with an equal volume of CFA using manual emulsion method or POWER emulsification method. On day 0, 8-10 week old male C57BL/6 were immunized subcutaneously with 100μg of mBSA in CFA. On day 21, both knees were shaved and 100 µg of mBSA was administered intra-articularly in the right knee joint. Left knee joints received a vehicle control injection. From day 21 onwards, mice were treated daily with an IP injection of dexamethasone (2mg/kg) or vehicle. Knee swelling was monitored daily using digital calipers.

For IVIS imaging, mice received an intravenous injection of 4 nmol ProSense 750 FAST imaging probe (PerkinElmer) one day after intra-articular injection of mBSA. Mice were imaged 20 hours post intravenous injection using the IVIS Spectrum (Perkin Elmer, Waltham, Massachusetts, USA) with an excitation wavelength of 745 nm and an emission wavelength of 800 nm. Images were analyzed using Living Image 4.7 software (Perkin Elmer) to obtain the average fluorescence intensities of a circular region of interest encompassing the knee joint.

Articular nociception was evaluated using a dynamic weight bearing apparatus (Bioseb, France) as previously described (27). For testing, the mouse was placed in the chamber and allowed to move freely within the apparatus for period of 4 minutes. All movements were filmed and validated by the experimenter in accordance with the position of the mouse on the device, indicating which paw corresponded to the set of pixels recognized by the sensors. The software analyzed each acquisition and data of the weight distribution of each paw was used for subsequent analysis. The results are expressed as percentage weight ratio of the rear paw (left) over the ipsilateral rear paw (right).

At the end point of experiment, knee joints were harvested and fixed in 4% neutral-buffered formalin for 24 hours. Knee joints were embedded in paraffin and cut to 5µm sections. Sections were stained with safranin O/fast green. Histopathologic changes within the joint were assessed by blind scoring of the slides. A scoring system of 0-4 was assessed for synovial lining thickness, sub-synovial inflammation, cartilage matrix loss, chondrocyte death, bone marrow proliferation and bone erosion, giving a total score out of 24.

### Delayed Type Hypersensitivity

bCol2 was prepared from calf nasal cartilage by pepsin digestion and was purified as previously described (28). Mice housed at the Karolinska Institute were immunized with 100μg/ml of bCol2 emulsified 1:1 in CFA (Difco, Detroit, MI, USA) at the base of the tail. On Day 10 after immunization, the mice were challenged with bCol2 (dissolved in 0.1 M acetic acid) mixed with PBS and injected intradermally into the right ear (10μg in 0.02 M acetic acid per mouse). The left ear was injected with the vehicle (PBS/0.02 M acetic acid) alone and used as a control. Ear thickness was measured 24, 48 and 72 hours after challenge and the difference of ear thickness between right and left ear was calculated.

### Statistical Analyses

Data are presented as the arithmetic mean ± SEM. Statistical analysis was performed using GraphPad Prism 8 software. For clinical scoring data with immunization group and time as independent variables, a two-way repeated-measures ANOVA with Tukey *post-hoc* analysis was performed. For data in which immunization groups with treatments were the independent variables, one-way ANOVA with Tukey *post-hoc* analysis was performed. Probability values (P) of less than 0.05 were considered significant.

## Results

### POWER-Kit™ Produces Stable and Homogenous Emulsions

Several different techniques exist to produce water-in-oil emulsions, for example with syringes (29), by sonication (30), and by vortexing (31). However, it has been difficult to develop a fast, reliable, and standardized method of preparation of water-in-oil emulsions that is high enough quality to induce disease in pre-clinical autoimmune models such as EAE, CIA and AIA.

**Figure 1:**
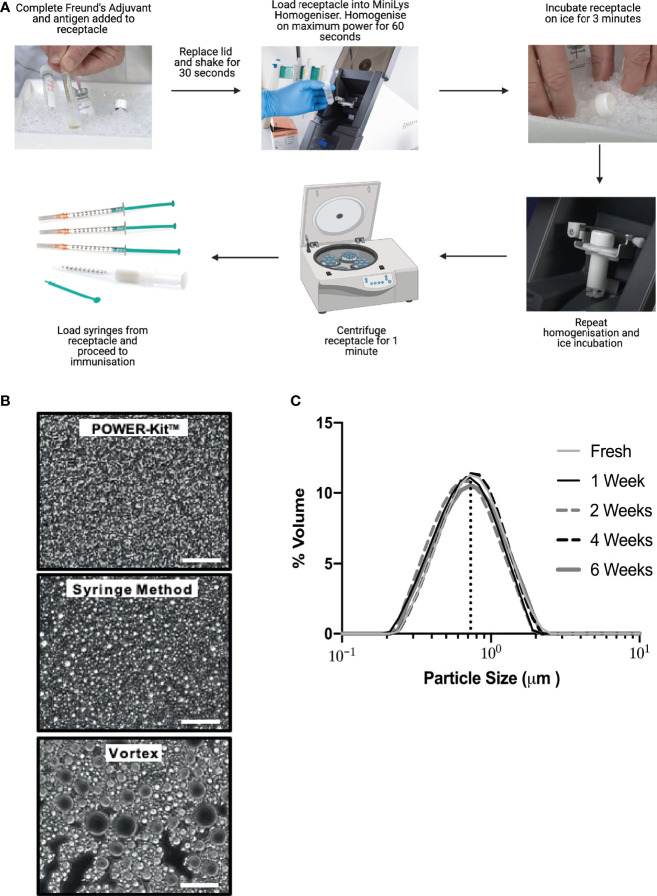
Characterization of emulsions produced using the POWER-Kit™ method. **(A)** Schematic of the process of emulsion preparation using POWER-Kit™. **(B)** Representative images from three different preparations methods; POWER-Kit™ method, traditional syringe method, and with a standard Vortex. A small amount of emulsion was placed on a glass slide and observed under a phase contrast microscope (400x). The scale bar in each Figure represents 50 µm. Shown is one representable preparation of each method from >15 experiments. The average D [4, 3] (the volume weighted mean) was measured with a Mastersizer 2000 and for the samples shown in panel B were 0.7 µm for the POWER-Kit, 1.4 µm for the syringe method, and finally 5.4 µm for the vortex method (data not shown). **(C)** Stability of emulsion over time was measured using the Mastersizer 2000. One emulsion of CFA/PBS was prepared with the POWER method and particle size determined using laser diffraction over time. One sample was analyzed immediately and then the emulsions were stored at +4°C. These samples were analyzed 1, 2, 4 and 6 weeks after preparation. The average D [4, 3] volume weighted mean for all samples were 0.73µm, with a standard deviation of +/- 0,03.

To introduce a standardized method of antigen-emulsion preparations to induce EAE, CIA, AIA and other pre-clinical models dependent on CFA/autoantigen immunization, we have developed a technology using a standard tissue homogenizer. The emulsions are generated by homogenizing within a purposely designed container, guaranteeing high quality and eliminating person-to-person variations, which are then easily transferred to syringes used for immunization (**Figure 1A**).

We set out to compare antigen-emulsions prepared with the POWER-Kit™ method with the traditional syringe method and emulsions produced through vortexing. Emulsions were prepared as described in the Materials and Methods section. All methods produced water-in-oil emulsions, determined by the drop-test method (data not shown). The vortex method generated emulsions with low viscosity, whereas the POWER-Kit™ and the syringe method generated emulsions of higher viscosity (data not shown). To assess the homogeneity and quality of the emulsions, a small drop was placed on a glass-slide and observed by microscopy. As shown in **Figure 1B**, emulsions produced by POWER-Kit™ displayed a uniform and small particle, the syringe method a more heterogenous emulsion, and the Vortex method an irregular particle size appearance. By using laser diffraction measures, the particle size distributions in **Figure 1B** were analyzed. The POWER-Kit™ method generated small, uniform and homogenous particles (0.7 µm), the syringe method produced slightly larger particles on average (1.4 µm), and the vortex method produced particles that were substantially larger (5.4 µm) and heterogenous. Therefore, the POWER-Kit™ method generated emulsions resulted in reproducible, homogenous water in-oil emulsion that outperformed both the syringe and vortex methods. However, both the POWER-Kit™ and syringe methods induced disease successfully (see **Figure 3**).

**Figure 2:**
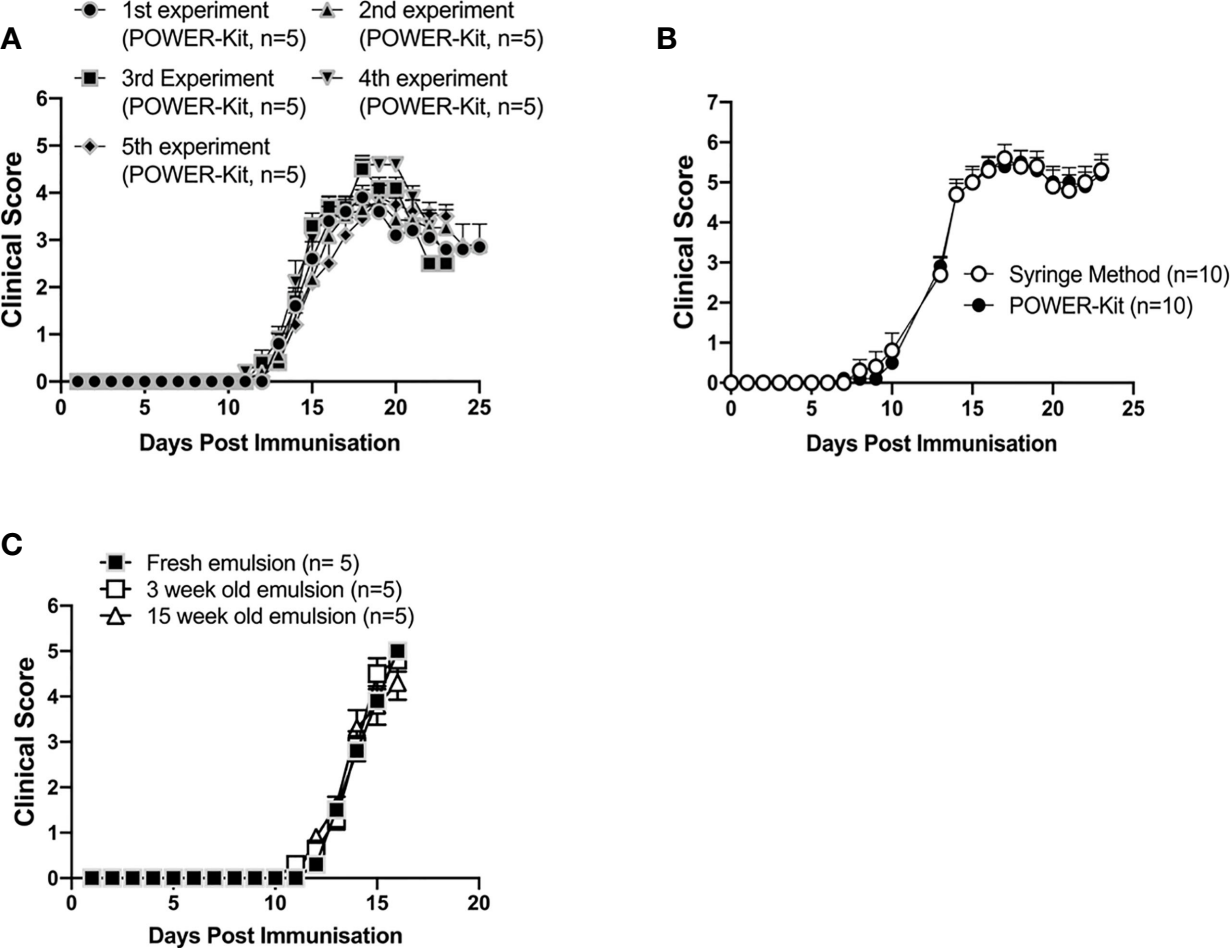
Experimental autoimmune encephalitis (EAE) induction in mice immunized using the POWER-Kit™ method. **(A)** Consistent induction of EAE by the POWER-Kit™ method. Emulsions containing CFA and 50µg MOG_35-55_ peptide/mouse were prepared with POWER-Kit™ at different time points and injected into C57BL/6JRj mice (5/group). Mice received 80ng of pertussis toxin on the same day and 24 hours later. Mice were scored for signs of disease according to the protocol at Lund University. **(B)** Induction of EAE with emulsions prepared by the POWER-Kit™ method or standard 2-syringe method, both prepared and used at Redoxis (Lund, Sweden). The emulsions contained CFA and 200µg MOG_35-55_ peptide/mouse were prepared in parallel and injected into C57BL/6 mice (10/group). Mice were scored for signs of disease according to the protocol at Redoxis. **(C)** EAE induction of stored emulsion. Emulsions containing CFA and 50µg MOG_35-55_ peptide/mouse were prepared with POWER-Kit™ and stored at +4°C for 3 or 15 weeks. Newly prepared and the stored emulsions were injected the same day into C57BL/6JRj mice (5/group). Mice received 80ng of pertussis toxin on day 0 and 24 hours later. Mice were scored for signs of disease according to the protocol at Lund University.

**Figure 3:**
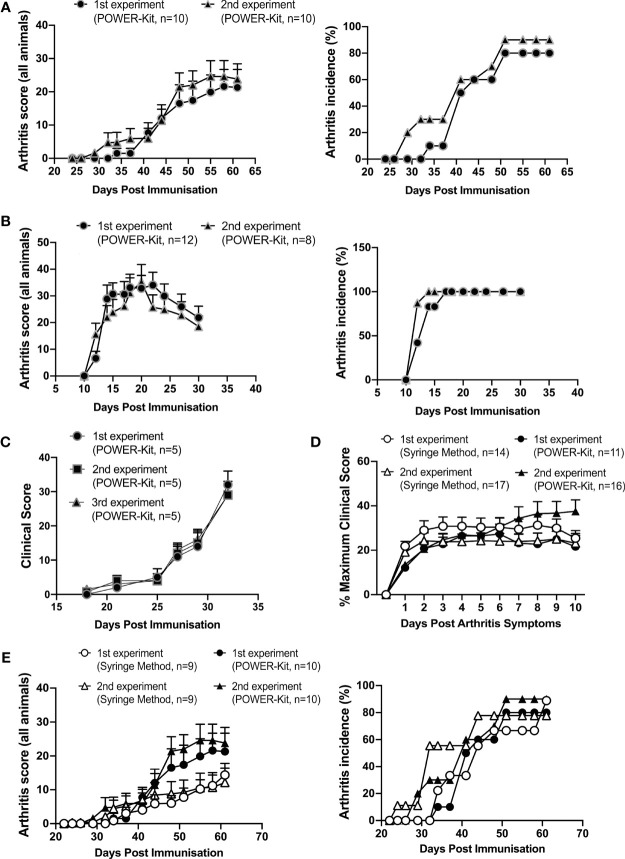
Experimental polyarthritis models are reproducibly induced by immunization using the POWER-Kit™ method. **(A)** Comparison of mean clinical score and incidence (percentage of affected mice) of two independent experiments of CIA in QB mice, using the POWER-Kit™ method. To induce CIA, animals were immunized with 100 µg of rCol2 in CFA on day 0 and boosted on day 35 with 50 µg of rCol2 in IFA. Mice were scored for signs of disease according to the protocol at the Karolinska Institute. **(B)** Comparison of mean clinical score and incidence of two independent experiments of hGPI peptide 325-339-induced arthritis (GPIA) in B10.Q mice. To induce GPIA, animals were immunized with 10 µg of hGPI peptide 325-339 in CFA. hGPI experiments were scored for signs of disease according to the protocol at the Karolinska Institute. **(C)** Induction of CIA using chicken Col2 (cCol2). Mice (10/group) were immunized with 100μg/mouse cCol2 emulsified with CFA, according to Redoxis protocol for cCIA. Mice were given a booster injection of 100μg/mouse cCol2 in IFA on day 21, and thereafter scored for clinical signs of disease. **(D)** Comparison of CIA induction in DBA/1 mice using the syringe and POWER-Kit™ method of immunization. DBA/1 mice were immunized with 200µg of bCol2 in CFA. Mice were monitored daily for disease and scored according to the protocol at the Kennedy Institute of Rheumatology. **(E)** Comparison of mean clinical score and incidence of two independent CIA experiments performed using the manually prepared emulsions with two independent CIA experiments done using the POWER-Kit™ device in QB mice. Mice were scored according to the protocol at the Karolinska Institute.

To test the stability of the emulsions, samples produced by POWER-Kit™ were stored at +4 degrees Celsius for the specified time before the particle size was analysis by laser diffraction. Newly prepared emulsion was compared to samples stored for 1, 2, 4 or 6 weeks. There was no difference in average particle size across samples (**Figure 1C**) and no signs of phase-separation in any of the samples (data not shown). Thus, emulsions prepared with the POWER-Kit™ method can be stored for at least 6 weeks in the fridge without compromising the quality, in terms of the size of the water-in-oil particles or phase-separation.

### POWER-Kit™ Emulsions Reproducibly Induce Experimental Autoimmune Encephalitis in Mice

One of the most used pre-clinical models for the human disease MS is EAE. Several FDA approved drugs for MS have been developed from results initially discovered using the murine EAE model (namely Natalizumab, Glatiramer Acetate and Mitoxantrone). There are a number of different murine EAE models (32) and the quality of the emulsion is considered an important factor to generate reproducible results. It must be a water-in-oil emulsion, containing small homogeneous particles to induce reproducible results. The method to prepare the antigen/CFA emulsion to study disease mechanisms and potential immunotherapies using the EAE model varies. A common method employs 2 syringes and a three way ‘T’ connector to mix the emulsion (29). The quality of the prepared emulsion is likely to differ each time and it would be challenging to standardize this method since it depends on a number of factors controlled by the person preparing the emulsion.

The POWER-Kit™ method was developed to assist in the standardization of antigen-emulsion preparations for the induction of autoimmune disease models. To test the reproducibility and to compare with the commonly used syringe method, a number of experiments were carried out. Over a period of six months, five separate EAE experiments were performed (**Figure 2A**). C57BL/6J mice were immunized with the MOG_35-55_ peptide and scored for clinical disease. The emulsion was prepared using the POWER-Kit™ method on the day of immunization. All the reagents were stored as stock solutions to ensure consistency (MOG_35-55_ peptide 10mg/ml and pertussis toxin 200μg/ml at -20°C).

Mice developed a similar disease-pattern in all experiments (**Figure 2A**). Maximum disease-score ranged from 4 to 6, and the recovery rate differed slightly in the different experiments, which could reflect season variability, depending on which month of the year the mice were born as described by Álvarez-Sánchez et al. (33). A direct comparison between emulsions prepared with the syringe method to the POWER-Kit™ method was performed (**Figure 2B**). There was no statistical difference in disease-score at any time-point throughout the whole experiment, indicating that the POWER-Kit™ method can perform as well as the syringe method in terms of clinical efficacy.

We have shown in **Figure 1C** that the quality of the emulsion in terms of particle size does not change over a period of six weeks. However, whether emulsions containing the MOG_35-55_ peptide also can be stored at +4°C for a period of time and still induce comparable disease is not known. Subsequently, emulsions with MOG_35-55_ peptide were prepared with the POWER-Kit™ method and stored in the fridge at +4°C for 3 or 15 weeks. Then, a new emulsion was prepared and C57BL/6J mice were immunized with either preparation. The stored emulsions showed no phase separation. The fresh, 3-week-old and 15-week-old emulsions induced similar disease-scores throughout the experiment (**Figure 2C**). These results show that emulsions prepared with the POWER-Kit™ method containing MOG_35-55_ peptides can be stored for at least 15 weeks and still induce EAE with comparable disease-scores to emulsions prepared at the time of the experiment.

### POWER-Kit™ Emulsions Reproducibly Induce Disease in Mouse Models of Polyarthritis

For preclinical RA research, polyarthritis models remain the models of choice for many due to their similarity to human disease. However, models such as CIA and glucose-6-phosphate isomerase peptide arthritis (GPIA) remain difficult to induce consistently, with many laboratories experiencing low disease penetrance and variable results across experiments (personal communications). To test the ability of POWER-Kit™ emulsions to induce polyarthritis murine models of disease, we induced CIA and GPIA across three research institutes. As shown in **Figure 3A**, CIA was reproducibly induced across two experiments performed at the Karolinska Institute. The mean clinical score of CIA mice was comparable across the two experiments (**Figure 3A** left panel), with similar, high disease penetrance across the two repeats (**Figure 3A** right panel). This result was also reproduced in type II chicken collagen induced arthritis mouse model performed at Redoxis, Sweden (**Figure 3C**). Over three independent experiments, POWER-Kit™ emulsions were able to induce disease with very high reproducibility. Additionally, GPIA was also reproducibly induced over two experiments at the Karolinska institute (**Figure 3B**). The mean arthritis score of GPIA mice was similar over the 30 days of monitoring across the two repeats (**Figure 3B** left panel), with 100% disease penetrance in both repeats (**Figure 3B** right panel).

POWER-Kit™ method was also compared with the traditional syringe method, which is currently the gold-standard for emulsion preparation. In the bovine collagen II (bCol2) CIA method performed at the Kennedy Institute of Rheumatology, disease induction in experiments using the POWER-Kit™ followed a similar pattern to experiments using the traditional syringe method. There was no significant difference in disease scores over time between the POWER-Kit™ method and syringe method. In experiments performed at the Karolinska Institute, the average clinical score in mice immunized with POWER-Kit™ was higher than mice immunized with syringe method emulsion, although not statistically significant (**Figure 3E** left panel). The apparent higher clinical scores using the POWER-Kit™ may be due to the more homogenous emulsion compared to that made by the syringe method (**Figure 1B**). Disease penetrance was similar between the two methods (**Figure 3E** right panel).

### Consistent Induction of Delayed-Type Hypersensitivity Model in Mice Using POWER-Kit™ Emulsions

The delayed-type hypersensitivity (DTH) is a quick and non-invasive model based on the induction of local cellular immune responses. In mouse models, during the sensitization phase, mice are subcutaneously exposed to collagen emulsified with CFA. Following exposure, during the elicitation phase, mice are re-exposed to collagen used for sensitization by dermal injection into the ear, which results in the trafficking of collagen-specific T lymphocytes to the site of antigen deposition and the subsequent production of proinflammatory cytokines. The DTH response can be evaluated by the measure of ear thickness (34).

**Figure 4:**
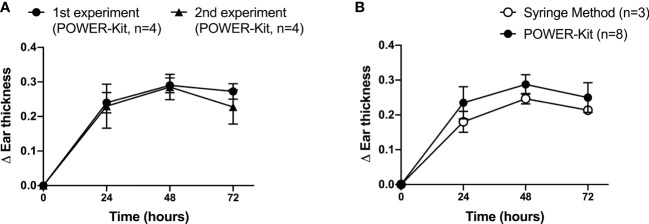
High reproducibility of delayed-type hypersensitivity (DTH) response induced by immunization using the POWER-Kit™ method. **(A)** Comparison of two independent DTH experiments using the POWER-Kit™ method. To induce DTH, HLA-DRB1*0401.hIi.cia9i mice were immunized with 100 µg of bCol2 in CFA on day 0 i.d. On day 8, 10μg of bCol2 in PBS was injected intradermally into the left ear and PBS to the right ear. The ear thickness was measured at 24, 48, and 72h. **(B)** Comparison of two independent delayed-type hypersensitivity (DTH) experiments performed using the manually prepared emulsions or using the POWER-Kit™ device.

DTH is useful in the initial steps of preclinical development of new immunomodulatory agents in RA. At Karolinska Institute, the ability of POWER-kit™ emulsions to induce a reproducibility inflammatory response in humanized mice was tested. As shown in **Figure 4A**, the mean ear thickness increment was comparable across two independent experiments. As shown in **Figure 4A**, the ear thickness increased at 24 h after intradermal injection and reached the peak at 48 h in both experiments. We can observe a high reproducibility between both experiments with graphs almost overlapping. POWER-kit™ method was also compared with the traditional syringe method. **Figure 4B** shows the mean of ear thickness increment in mice immunized with POWER-kit™ was larger than mice immunized with syringe method emulsion, although not statistically significant, using the two-way repeated-measures ANOVA with Tukey *post-hoc* analysis.

### Dexamethasone Treatment in Murine Monoarthritis Is Unaltered in Mice Immunized With POWER-Kit™ or Syringe Method

**Figure 5:**
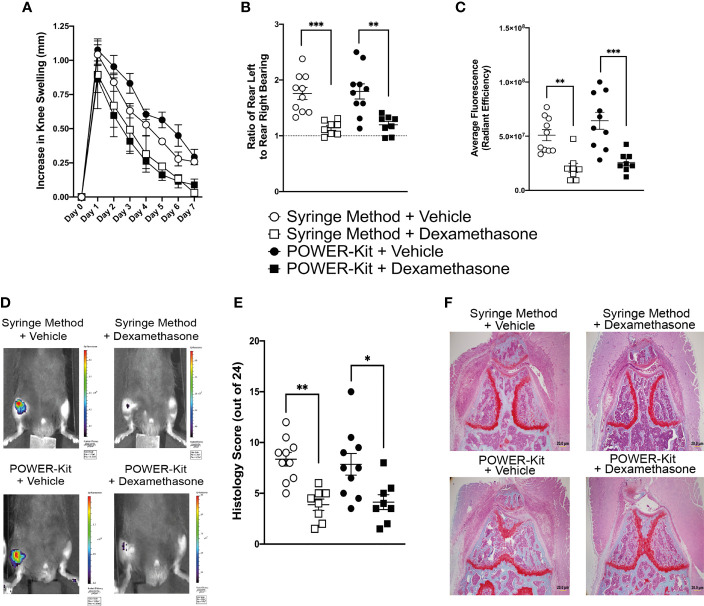
Treatment of experimental monoarthritis in mice is unaltered by immunization using the POWER-Kit™ method. **(A)** Knee swelling of antigen-induced arthritis (AIA) mice, induced using either the syringe method or POWER-Kit™ method and treated with either vehicle or dexamethasone. **(B)** Pain assessment of AIA mice, showing ratio of weight bearing of contralateral knee over ipsilateral knee. **(C)** IVIS imaging quantification of cathepsin activity within the arthritic knee. **(D)** Representative IVIS images of cathepsin activity within the arthritic knee. **(E)** Quantification of histological scoring of safranin O/fast green stained knee sections. Knee joints were scored from 0-4 on synovial hyperplasia, subsynovial infiltration, cartilage matrix staining, chondrocyte death, bone marrow hypercellularity and bone erosion. **(F)** Representative images of AIA knees stained with safranin O/fast green. *p< 0.05, **p<0.01, ***p<0.001.

Having established that the POWER-Kit™ method successfully induces disease in a range of models, we moved on to assess whether induction of disease using the POWER-Kit™ method alters response to an established treatment regime. We employed the antigen induced arthritis (AIA) model with a daily dexamethasone treatment regime. Both vehicle and dexamethasone treatment were administered to mice immunized using the syringe method or POWER-Kit™ method. As shown in **Figure 5A**, knee swelling of syringe method and POWER-Kit™ immunized mice treated with vehicle were similar across all days and not statistically significant, indicating that the POWER-Kit™ method successfully induces AIA. In both the syringe method and POWER-Kit™ groups, mice treated with dexamethasone showed a reduction in knee swelling. However, the reduction in POWER-Kit™ immunized mice was statistically significant from day 2 onwards, compared to the syringe method group only reaching statistical significance on day 5 and day 7. Pain analysis of AIA mice was performed using a dynamic weight bearing device (**Figure 5B**). Mice immunized with syringe method or POWER-Kit™ and treated with vehicle control experienced high levels of pain, with an average ratio of rear leg weight bearing of 1.76 and 1.8 respectively. Mice treated with dexamethasone had reduced pain levels compared to the vehicle treated mice in both the syringe method and POWER-Kit™ immunized mice, with a ratio of 1.14 and 1.19, respectively. To further assess inflammation levels in the mice, IVIS imaging was performed using an imaging probe that cleaves in the presence of cathepsins and emits a near-infrared fluorescence. Quantification of the cathepsin signal in the AIA knee is shown in **Figure 5C** with representative IVIS images shown in **Figure 5D**. Vehicle treated mice showed levels of high cathepsin activity in the AIA knee in both immunization groups, and as expected dexamethasone significantly reduced the cathepsin levels in both syringe method and POWER-Kit™ immunized groups. At day 7, knee joints were harvested and processed for histological analysis. Safranin O/Fast Green stained sections were blindly scored to assess disease markers such as synovial lining expansion, subsynovial inflammation and cartilage damage. Vehicle treated mice from both immunization groups showed high disease activity with substantial cell infiltration, as shown from the representative images in **Figure 5F**. Mice treated with dexamethasone showed a significant reduction in disease activity compared to vehicle treated mice in both immunization groups as shown in **Figure 5E**.

## Discussion

The induction of experimental autoimmune disease by the emulsification of antigens with immunostimulants has proved successful to replicate multiple human diseases in animals. However, in some models, the induction of disease is difficult to achieve and reproducibility between experiments is poor. There is currently no standardized method to prepare antigen emulsions, and manual mixing is the gold standard for most laboratories. Manual mixing is time consuming, results in excessive loss of material, difficult to compare between laboratories and could potentially cause repetitive strain injury. Herein, we have tested a novel automated homogenization method for the preparation of antigen emulsions for the induction of experimental autoimmune disease, namely POWER-Kit™ method.

The POWER-Kit™ method induces disease consistently in a number of autoimmune models and shows high reproducibility between experimental repeats. Furthermore, the POWER-Kit™ method has now been validated across multiple independent research institutes across countries with insignificant variation in results. Our studies have also demonstrated that POWER-Kit™ induces disease as well as, and in some cases better than, the method of manual syringe mixing. Additionally, animals treated with therapeutic agents respond efficiently after immunization with POWER-Kit™ to the same degree as those immunized by manual syringe method. We are therefore confident that POWER-Kit™ is equivalent to the current gold standard with regards to disease induction.

We also tested induction of DTH by the POWER-Kit™ method and compared it to the manual syringe method. Both methods induce highly reproducible results, showing that more general immune responses are also induced by the POWER-Kit™ method, to the same degree as the syringe method. These results indicate that the POWER-Kit™ method can be used for several immunization schemes that rely on water-in-oil emulsions. For example, generation of cancer vaccines, production of mono- and poly-clonal antibodies to generate diagnostic tools for medical purposes, and monoclonal antibodies treat human diseases are potential avenues for adoption.

There are additional significant benefits to using POWER-Kit™, such as saving time and reduced loss of expensive reagents. The time to prepare emulsions is reduced using POWER-Kit™ compared to the manual syringe method. For example, one laboratory has estimated that it takes approximately 1.5 hours to prepare a MOG_35-55_ emulsion (35). The process of preparing emulsions and loading syringes takes approximately 30 minutes with the POWER-Kit™ method, which is a large reduction compared to the manual syringe method. With regard to the manual syringe method, preparing and loading syringes with emulsion for the injection of animals can be troublesome and problematic. This is often associated with large losses of reagents as well as the introduction of air bubbles within the syringes. The POWER-Kit™ method has been designed and optimized so that syringes are easily loaded from the cartridge in which the emulsion is homogenized, which eliminates the introduction of air bubbles and the loss of any material. With the POWER-Kit™ method, one has to prepare an extra 20% of the total volume needed for loading all syringes and for QC. This is a huge difference to the standard 2-syringe method where 50-100% extra has to be prepared (36, 37). The reduction in loss of reagents results in the POWER-Kit™ method being more cost effective long-term.

Using the method presented here, we show that the emulsions generated are stable over a long period of time. POWER-Kit™ emulsions made with the MOG_35-55_ peptide could be stored up to 15 weeks and still induce EAE to a similar degree as freshly made emulsions, indicating that the emulsions are functionally stabile over a long period of time. Whether POWER-Kit™ emulsions containing other antigens can be stored for a long period of time, needs to be determined for each specific antigen.

The ability of manual emulsions to induce disease after prolonged storage has not been assessed in this study. However, protocols using the standard manual methods suggest making up fresh emulsions for each immunization.

The principles of the 3Rs (Replacement, Reduction and Refinement) were developed over 60 years ago and it is still important to ensure that high ethical procedures are applied and that the number of animals used in scientific experiments is kept to a minimum. Therefore, with the use of POWER-Kit™ there is a great opportunity to incorporate one of the frameworks (Reduction) for more humane animal research, reduction. Firstly, we have found that there is close to 100% induction of disease in the different autoimmune models tested when using POWER-Kits™. Therefore, fewer animals can be used to achieve statistical difference between treatment groups, particularly in hard-to-induce models. Secondly, since the emulsion is prepared with a technology independent of human influence, results both within (emulsions prepared by different researchers) as well as between different laboratories can easier be compared.

Cancer vaccines. Vaccine adjuvants, such as Montanide™ ISA 51, has shown to be a promising adjuvants for cancer vaccines (19). A standardizes protocol to prepare water-in oil emulsions in the clinic would have a huge impact, not only in clinical trials but also when treating patients with cancerous diseases. Preliminary results have shown that the POWER-Kit™ method generates emulsions with Montanide™ ISA 51 that are equivalent or even more homogenous compared with the manual syringe method (data not shown).

Although induction of other autoimmune disease, by a water-in-oil emulsions of autoantigen and CFA, have not been tested with the POWER-Kit™ method, we anticipate that the method can be readily adopted for induction of a number of different autoimmune disease models. For example, experimental allergic neuritis (EAN), experimental autoimmune thyroiditis (EAT), autoimmune uveitis (EAU), Guillain–Barre´ syndrome (GBS), myasthenia gravis (MG), and systemic lupus erythematosus (SLE) (38). In summary, we have validated the use of the POWER-Kit™ method to induce autoimmunity disease models and we are currently validating its use for other indications, such as mono- and poly-clonal antibody production *in vivo*. The lower cost, saved time, robust and reproducible disease induction and ease of the POWER-Kit™ method provide significant benefits over the traditional syringe method, and offer a well overdue standardization of a method vital to biomedical research.

## Data Availability Statement

The raw data supporting the conclusions of this article will be made available by the authors, without undue reservation.

## Ethics Statement

The animal study was reviewed and approved by Animal Ethics Committee, Lund-Malmö, Sweden, Ethical Review Process Committee, Oxford, England, Stockholms Norra Djurförsöksetiska Nämnd, Stockholm, Sweden.

## Author Contributions

Conceptualization: LT, RH, RW, and BB; Methodology: LT, LR-C, VU, HB, FC, and BB; Investigation: LT, LR-C, VU, FC, and BB; Supervision: RH and RW; Writing—original draft: LT and BB; Writing—review and editing: All authors. All authors contributed to the article and approved the submitted version.

## Funding

This work was supported by grants from: The Knut and Alice Wallenberg foundation (grant number 2019-0059) (RH); The Swedish Medical Research Council (grant numbers 2017-06014, and 2019-01209)(RH).

## Conflict of Interest

BTB Emulsions AB has submitted a patent application for the use of a device and method for preparing emulsions for immunization and animals and humans (European patent application number: EP3836884A1). BB is the CEO and founder of the company, and a shareholder in BTB Emulsions AB.

The remaining authors declare that the research was conducted in the absence of any commercial or financial relationships that could be construed as a potential conflict of interest.

## Publisher’s Note

All claims expressed in this article are solely those of the authors and do not necessarily represent those of their affiliated organizations, or those of the publisher, the editors and the reviewers. Any product that may be evaluated in this article, or claim that may be made by its manufacturer, is not guaranteed or endorsed by the publisher.
